# Effect of augmenting cholinergic function on gait and balance

**DOI:** 10.1186/s12883-015-0523-x

**Published:** 2015-12-23

**Authors:** Martina Mancini, Brett W. Fling, Anne Gendreau, Jodi Lapidus, Fay B. Horak, Kathy Chung, John G. Nutt

**Affiliations:** Parkinson Center of Oregon, Department of Neurology, Oregon Health & Science University, 3181 S.W. Sam Jackson Park Rd., Portland, OR 97239-3098 USA; Biostatistical Research Program, OHSU, Portland, OR USA; Veterans Affairs Portland Health Care System, Portland, OR USA

**Keywords:** Parkinson’s disease, Cholinergic system, Gait & balance, Attention

## Abstract

**Background:**

Impaired mobility and falls are clinically important complications of Parkinson’s disease (PD) and a major detractor from quality of life for which there are limited therapies. Pathological, neuroimaging and clinical evidence suggest that degeneration of cholinergic systems may contribute to impairments of balance and gait in PD. The proposed trial will examine the effects of augmentation of the cholinergic system on balance and gait.

**Design:**

The study is a single-site, proof of concept, randomized, double-blind, cross-over trial in patients with PD. Each treatment period will be 6 weeks with a 6-week washout between treatments for a total of 18 weeks for each subject. Donepezil in 2.5 mg capsules or identical appearing placebo capsules will be increased from two per day (5 mg) to four capsules (10 mg) after 3 weeks, if tolerated. Subjects will have idiopathic Parkinson’s disease, Hoehn and Yahr stages 2 to 4. We anticipate recruiting up to 100 subjects for screening to have 54 enrolled and 44 subjects complete both phases of treatment. Dropouts will be replaced. As this is a crossover trial, all subjects will be exposed to both donepezil and to placebo. The primary outcome measures will be the root mean square of the mediolateral sway when standing and the variability of the stride duration when walking for two minutes. Secondary outcomes will be the computerized Attention Network Test to examine three domains of attention and the Short-latency Afferent Inhibition (SAI), a physiological marker obtained with transcranial magnetic stimulation as a putative marker of cholinergic activity.

**Discussion:**

The results of this study will be the most direct test of the hypothesized role of cholinergic neurotransmission in gait and balance. The study is exploratory because we do not know whether donepezil will affect gait, balance or attention, nor which measures of gait, balance or attention will be sensitive to drug manipulation. We hypothesize that change in cholinergic activity, as measured with SAI, will predict the relative effectiveness of donepezil on gait and balance. Our immediate goal is to determine the potential utility of cholinergic manipulation as a strategy for preventing or treating balance and gait dysfunction in PD. The findings of this trial are intended to lead to more sharply focused questions about the role of cholinergic neurotransmission in balance and gait and eventually to Phase II B trials to determine clinical utility of cholinergic manipulation to prevent falls and improve mobility.

**Trial registration:**

This trial is registered at clinical trials.gov (NCT02206620).

## Background

Gait and balance problems occur throughout the course of Parkinson’s disease (PD), appearing within three years of diagnosis [1–3]. In PD, falls result in injuries [4], mortality, death, fear of falling [5] and decreased quality of life [6]. Although levodopa improves some gait parameters [7] levodopa does not improve, and may worsen, several types of balance control [8]. For example, postural sway area and sway velocity during quiet stance increase with levodopa in patients with moderate to severe PD [8]. In addition, the size of automatic postural responses to external perturbations decrease with levodopa, which could lead to falls from poor recovery from a slip or trip [9]. In general, as the disease progresses, levodopa is less efficacious for balance and it is assumed that non-dopaminergic systems are impaired later in the disease course [10]. Deep Brain Stimulation (DBS) also is generally not effective for reducing falls [11].

Although the dopaminergic system is preferentially affected in PD, there are also changes in the basal forebrain cholinergic complex that provides cholinergic innervations of the cortex. In addition, the pedunculopontine nucleus (PPN), part of the midbrain locomotor region (MLR), has a prominent cholinergic projection to the thalamus and it degenerates in PD [12]. PET imaging with [11C]PMP, a substrate for acetylcholinesterase, identifies the integrity of cholinergic innervation. Loss of cortical cholinergic markers in PD representing degeneration of the basal forebrain nuclei is associated with slower gait [13] and impairments in working memory, attention and executive function [14]. Loss of thalamic [11C]PMP, representing loss of PPN cholinergic innervation of the thalamus, is related to falls in PD [15]. In contrast, the extent of dopaminergic denervation of the striatum evaluated by PET imaging was not correlated with falls [15]. Finally, neurotoxin lesioning of the cholinergic neurons of the PPN induced gait and postural abnormalities in monkeys [16].

The most direct test of the role of cholinergic systems in balance and gait to date are three small clinical trials [17–19]. In two randomized, double-blind, crossover clinical trial of a cholinesterase inhibitor, donepezil, our group found that donepezil reduced falls in patients with PD [17] and decreased mean sway velocity in the Sensory Organization Test [19]. An open trial with another cholinesterase inhibitor, galantamine, also reported decreases in falls and freezing of gait (FoG) [18]. But these small clinical trials are not conclusive.

The effects of manipulation of the cholinergic system could be mediated by at least two general mechanisms, altering attention via effects on cortical and subcortical cholinergic systems or altering balance and gait circuitry via effects on brainstem locomotor cholinergic circuits. In addition to balance and gait measures, short-latency afferent inhibition (SAI), a physiological index of central cholinergic function (ref) will be measured to determine if the deficits in balance and gait correlate with abnormalities of the SAI and if SAI is altered by donepezil as a measure of drug efficacy. Moreover, the attention network test (ANT) will be administered to determine if changes in gait and balance are mediated by changes in attention. This study will be the most direct test of the role of cholinergic systems in gait and balance. We hypothesized that measures of gait and balance will improve in response to cholinergic augmentation with a cholinesterase inhibitor, donepezil.

## Methods/Design

**Fig. 1:**
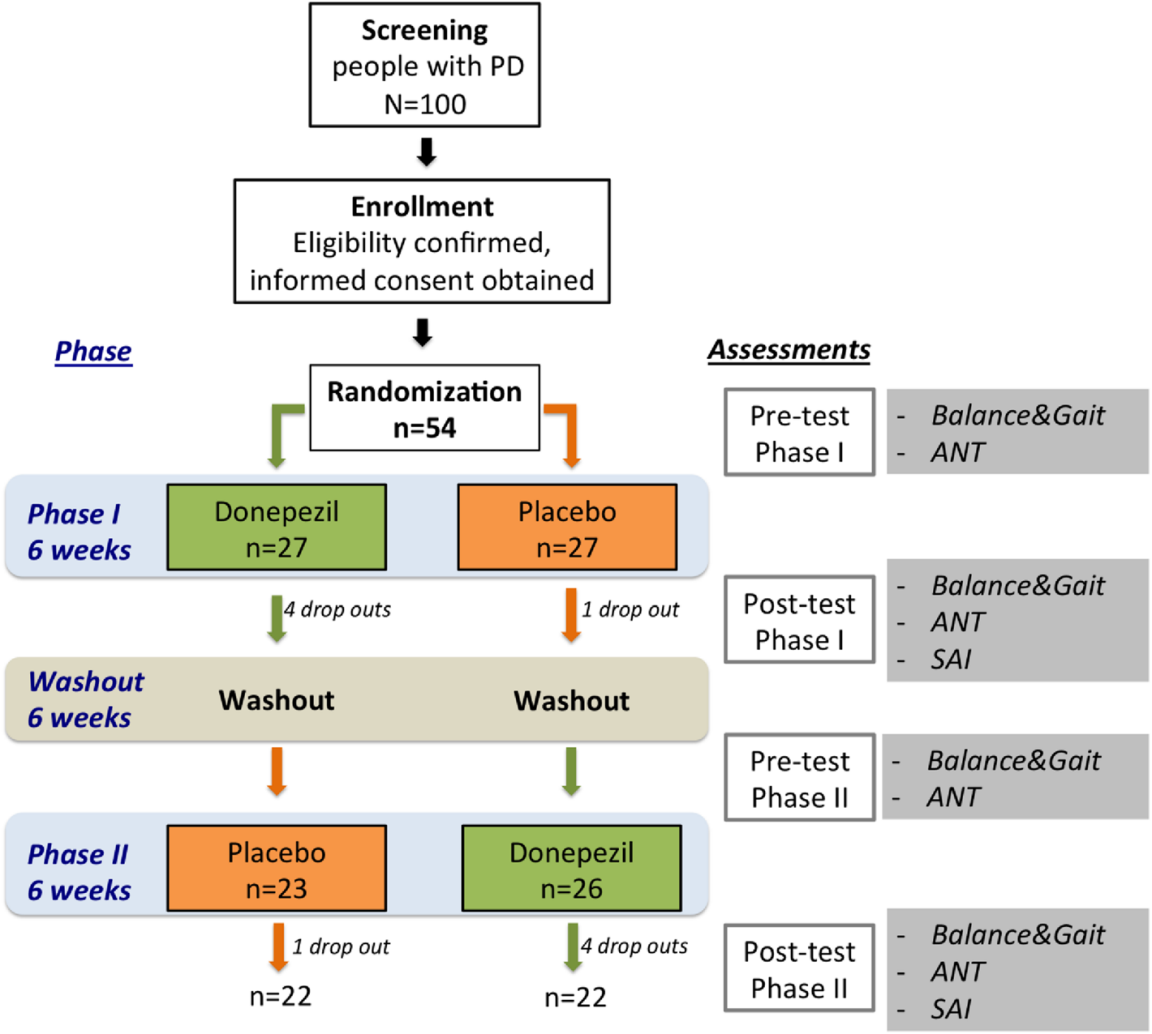
Study design and outcomes measures for each phase of the study

The study is a single-site, proof of concept, randomized, double-blind, cross-over trial in patients with PD. Each treatment period will be 6 weeks with a 6-week washout between treatments for a total of 18 weeks for each subject. See Fig. 1 for protocol details.

### Participants

We anticipate recruiting up to 100 subjects for screening to have 54 enroll and 44 complete both phases of treatment. Dropouts will be replaced. As this is a crossover trial, all subjects will be exposed to donepezil and to placebo. Subjects will be 30 years or older, of either sex and any racial or ethnic origin. Women who are capable of child bearing must employ birth control. Pregnant women are not eligible to participate. The subjects will have idiopathic Parkinson’s disease as determined from history and exam and lack of history or physical findings that would suggest another diagnosis or a parkinsonism-plus syndrome.

Subjects may be Hoehn and Yahr stages II to IV. Subjects must be able to stand unassisted for a minute and to walk continuously for 2 min without assistance or assistive devices. The subjects must have a Montreal Cognitive Assessment (MOCA) of 23 or above and be judged to be able to appreciate the purpose of the research, give informed consent to participate, be able to cooperate with the testing and be compliant with taking the experimental medications.

Exclusion criteria include: significant tremor which would interfere with recording balance and walking; other factors affecting gait such as musculoskeletal disorders (particularly symptomatic hip, knee and lumbar osteoarthritis), uncorrected vision disturbance, vestibular problems or any other health problem judged to interfere with participation. Major depression, hallucinations or other psychiatric disturbances will be exclusions. Medical problems that might be worsened by donepezil are exclusion criteria and include tachycardia, bradycardia, arrhythmias, and peptic ulcer disease. Finally, use of anticholinergics for parkinsonism, bladder antispasmodics for urinary urgency or tricyclic antidepressants for depression are contraindications as is concurrent use of cholinesterase inhibitors for cognitive problems.

### Intervention

See Fig. 1 for study design and summary of the assessments. Subjects will receive the first study phase medications from the research pharmacy at the end of the baseline testing. The research assistant will call subjects weekly to ask about side effects or problems with the experimental medicines. They will also be asked about falls. These contacts will be captured in case report forms. If there are medical problems, the investigator will call the subject or the subject will be brought into clinic for evaluation. At 6 weeks, the subject will return to the clinic for repeat testing with the measures captured at baseline (Fig. 1) and will undergo the SAI testing. The subjects will then enter the 6-week washout period. Although the subjects will be on no experimental medications, they will continue to be called weekly to inquire about side-effects, problems and falls. This is partly done to keep the subject involved in the protocol, as well as to collect data about falls. At the conclusion of the 6 weeks, the subjects will return to the clinic for repeat testing and to have medications dispensed for the final 6-week phase of the protocol. The final 6-week phase will be conducted exactly as was the first 6-week phase with repeat testing at the end of the 6 weeks.

Cholinesterase inhibitors, such as donepezil, are standard of care for patients with PD who have cognitive impairments [20]. We chose donepezil, an FDA approved drug, because it is possible to create a placebo for the donepezil tablet and it requires only once a day dosing. It is recommended to start donepezil at 5 mg per day to reduce adverse effects on the gastrointestinal system. We will increase the dose to 10 mg/day after 3 weeks, if tolerated, because that is the schedule used in our pilot study and this titration was well tolerated [17]. Donepezil has a half-life of 70 h and it requires approximately 15 days to reach steady state. The maximum effects on cognitive measures occur within 3 weeks and loss of effect requires more than 3 weeks and less than 6 weeks. In our pilot study, there were more falls at 3 weeks and 5 mg/day of donepezil than at 6 weeks and 10 mg/day of donepezil and 10 mg was more effective than 5 mg on cognitive measures in PD [20]. Washout between phases will be 6 weeks to minimize carry-over of effects.

### Randomization and blinding

The Research Pharmacy at OHSU will be responsible for purchasing study medication and creating blinded capsules for each subject, maintaining and storing drug, randomizing to maintain blinding, dispensing medication, and checking compliance by returned capsule count.

### Sample size

We estimated the sample sizes required to detect differential changes in the primary outcome measures between donepezil and placebo, assuming a crossover design with 4 measurement time points (pre-post each treatment interval, see Fig. 1). We informed sample size and statistical power calculations based on preliminary data from ten PD subjects enrolled in a blinded pilot trial of donepezil vs. placebo who should be similar to patients recruited for this proposal [19]. In that study, sway velocity during donepezil treatment intervals decreased 2.35 units on average, while those on placebo increased 5.38 units, with a common standard deviation estimate of 9. We set power at 0.80 and level of significance at 0.025 rather than 0.05 since there are two primary endpoints (to control for multiple comparisons). Additionally, we assume no period effects will be observed in the crossover design (that is, we do not anticipate the order that patients receive treatment will affect the outcomes). Calculations were conducted using SAS v9.3 PROC POWER. Under these assumptions, a two-sided hypothesis test comparing sway velocity between donezepil and placebo would require 44 patients with complete outcome data. However, we anticipate that some patients may drop out of the study while on active treatment due to side-effects. Thus, we intend to recruit and enroll 54 patients to allow for up to 20 % drop out.

### Assessment procedures

Subjects will have been off medications overnight (at least 12 h washout). We will study the subjects when they have been without drug overnight and are “off,” because we have shown that sway while standing still is sensitive to dyskinesia when the subjects are “on” [21]. Further, some of the measures of standing and walking are sensitive to levodopa [8] and we wish to examine the effects of cholinergic manipulation without interference from other drugs as much as possible.

Parkinsonism and general cognition will be documented with the MDS Unified Parkinson’s Disease Rating Scale (MDS-UPDRS) and the Montreal Cognitive Assessment (MOCA) [22–24]. Subjects will also have the Activities of Balance Confidence (ABC) [25] as a measure of mobility limitation and the Parkinson’s Disease Questionnaire (PDQ-39) [26] as a measure of health-related quality of life.

### Primary outcome measures

Balance and gait kinematic performance will be recorded by small, body-worn, inertial sensors, Opals by APDM. The Opals consist of wireless, synchronized, triaxial accelerometers and gyroscopes that record body motion at 128 Hz. The sensors will be applied bilaterally to the feet and wrists as well as to the sternum and pelvis with Velcro straps. Inertial data is wirelessly transmitted with radio signals to a laptop that controls the protocol, allows comment on individual trials, automatically calculate some balance and gait metrics (Mobility Lab software, www.apdm.com) and store raw data for further analysis in Matlab.

To characterize postural sway, subjects will stand with feet together with hands at their hips looking straight-ahead at a fixed point in 4 different conditions: 1) eyes opened, 2) eyes closed, 3) eyes open standing on foam pad, 4) eyes closed standing on foam pad.

To characterize gait, subjects will be instructed to walk at their comfortable pace down a 20-m long hallway, turn around at each end and continue walking back and forth. The 180° turns are automatically detected and analyzed separately by Mobility Lab. Subjects will be instructed to walk continuously at their natural pace without talking or looking around. The 2-min walk will provide the gait variability measurements.

Subjects will also perform three more balance and gait trials with a cognitive, dual task paradigm, which consist of counting backwards by 3’s. Subjects will first be presented with the task in sitting to make sure they understand the instructions and record a baseline for 1 min. Subsequently, subjects will perform the gait and balance task with and without the dual task. The conditions repeated with dual task are standing with eyes opened on firm surface and foam pad, as well as walking up and down the hallway for 1 min. This will allow calculating the dual-task costs on balance and walking [(dual-task balance or gait performance minus single-task performance)/single task performance*100] as well as the cost of performance of the dual task [(dual-task counting – single task counting)/single task counting*100].

*The primary measure of balance* will be medial-lateral postural sway range during quiet stance because: 1) it is a predictor of future fall risk in subjects with PD, 2) it changes significantly with exercise and 3) it predicts falls in elderly subjects. In addition to medial-lateral sway range, we will examine other measures that may reflect changes in the postural motor control loop including: total sway amplitude (measured as root mean square eg; variability around the mean position) and jerkiness of sway (measured by differentiating the acceleration values of sway).

*The primary measure of gait stability* will be the coefficient variability of stride duration (time) during a 2-min walk, as a surrogate for fall risk based on the literature [27]. *Secondary gait outcome* measures include the following metrics and their coefficients of variability: stride length, cadence, double support time, arm swing amplitude, and mean trunk rotation. In addition, to determine the interaction between attention and gait, we will measure the change in lateral sway velocity and the percent change in the variability of stride duration during dual-tasks versus single tasks (ie; dual-task cost).

### Secondary outcome measures

#### Attention Network Test (ANT)

Attention will be tested with the Attention Network test, a 15-min, computerized test that examines the effects of cues and targets within a single reaction time task to provide a means of exploring the efficiency of the alerting, orienting, and executive control networks involved in attention [28, 29].

#### Short-latency afferent inhibition (SAI)

SAI will be performed using a modified version of protocol described previously in literature [30, 31]. Surface EMGs from the first dorsal interosseous muscle of the most involved arm will record motor evoked potentials (MEPs) from contralateral motor cortex transcranial magnetic stimulation (TMS). We will measure SAI by applying peripheral conditioning stimuli (electrical stimulation of the median nerve at the wrist) followed by central test stimuli (TMS of motor cortex). The intensity of the conditioning peripheral stimuli will be set at the value that evokes a twitch of the abductor pollicis brevis muscle. TMS will be performed using a figure-eight stimulating coil (external loop diameter = 9 cm) powered from a Magstim 200 magnetic stimulator. We will determine the resting motor threshold, which is defined as the lowest stimulus intensity capable of eliciting at least five MEPs with an amplitude of 50 μV or higher in 10 consecutive trials. All subsequent trials to evoke SAI will utilize an intensity of 120 % of resting motor threshold. The peripheral conditioning stimuli will precede the cortical TMS at 6 different interstimulus intervals (ISIs). We will determine the ISIs based on the latency of the N20 component of the somatosensory evoked potential (SSEP). To determine the N20 latency of the median nerve, we will attach the active and reference electrodes 3 cm behind C3 and C4 respectively (10–20 system). SAI will be randomly tested at 6 different interstimulus intervals, with a minimum of 10 trials at each interstimulus interval (from N20 in 1-ms increments until N20 + 5 ms), with 20 unconditioned (test) stimuli also delivered randomly The peak-to-peak amplitude of the conditioned motor evoked potentials at teach interstimulus interval will be averaged and expressed as a percentage of the averaged unconditioned motor evoked potential (baseline). To reduce variability, the conditioned responses will be combined across all interstimulus intervals and be expressed as the percentage of the unconditioned MEP as described by others [32, 33]. SAI requires no background EMG activity in the target muscles so visual feedback of the background EMG will be used.

### Statistical analysis

Our statistical hypothesis is that the change in outcomes between the week 0 and week 6 of the donepezil and placebo phases will differ. Specifically, we will test the hypotheses that augmenting cholinergic neurotransmission will improve sway and gait dual-tasking; attention and SAI will be secondary endpoints. We anticipate the clinical measures of PD severity (MDS-UDPRS), quality of life (PDQ-39) and balance confidence (ABC) will be less sensitive to drug effects but will examine them as well.

A linear mixed model will be used to analyze outcomes resulting from the crossover design with repeated measures (pre/post for each treatment). The model will include treatment effects, terms to assess period or carryover effects (which are not anticipated, but will be assessed and controlled for), and random effects for patients within treatments. The observations on each subject will be measures obtained at week 0 and 6 under the different treatment conditions (donepezil/placebo). Since in a typical experiment using repeated measures, two measurements taken at adjacent times may be more highly correlated than two measurements taken further apart, we will use Bayesian Information Criterion (BIC) to explore optimal covariance structure for the model. The main focus of the study is to determine whether the two treatments exhibit differential change over time, (i.e. time by treatment interaction effect). In crossover designs, missing data have much greater influence on the analysis than they would in a parallel-groups design especially when missing data are not at random, such as drop-outs due to the adverse effects of treatments. We have conservatively estimated sample size required for the trial assuming some patients drop out during the donepezil phase. Our primary analytic strategy will employ an intent-to-treat (ITT) approach, whereby all randomized patients are included in analyses, even if they drop out before completing both treatment phases. However, we will assess and report the impact of dropout by a) comparing demographic and clinical characteristics of those that dropout to those that complete, and b) re-analyzing outcomes models to only those that complete the trial.

## Discussion

We are particularly interested in determining whether gait and balance are directly influenced by cholinergic manipulation or whether changes in attention capacity are primarily responsible for changes in balance and gait. If, for example, we find that ability to balance and walk when dual tasking is altered out of proportion to changes during standing and walking without dual tasking, it would suggest that cholinergic systems mainly contribute to attentional capacity required for balance and gait. If Donepezil has opposite effects on specific attention domains within the Attention Network Test, this argument would be strengthened. Because of evidence linking executive dysfunction with gait disorders, we expect that the executive attention may be affected out of proportion to alerting and orienting aspects of attention, although Posner and colleagues have linked alerting attention to cholinergic function [28, 29].
